# Bioinformatics analysis of differentially expressed miRNAs in non‐small cell lung cancer

**DOI:** 10.1002/jcla.23588

**Published:** 2020-09-23

**Authors:** Hui Yu, Zhonghao Pang, Gang Li, Tianyi Gu

**Affiliations:** ^1^ Department of Cardiothoracic Surgery Affiliated Hospital of Jiangsu University Zhenjiang China

**Keywords:** biomarker, lung adenocarcinoma, lung squamous cell carcinoma, miRNAs, non‐small cell lung cancer

## Abstract

**Objective:**

Non‐small cell lung cancer (NSCLC) contains 85% of lung cancer. Lung adenocarcinoma (LUAD) and lung squamous cell carcinoma (LUSC) are the largest NSCLC subgroups. The aim of the study was to investigate the underlying mechanism in developing more effective subtype‐specific molecular therapeutic procedures.

**Methods:**

A total of 876 specimens were used in this study: 494 LUAD tissues (ie, 449 LUAD tissues and 45 matched normal tissues) and 382 LUSC tissues (ie, 337 LUSC tissues and 45 matched normal tissues). The miRNA sequencing data were processed using R. The differential expressed miRNAs between lung cancer and normal tissues were analyzed using the limma package in R. Gene expression, Western blotting, hematoxylin and eosin staining, and luciferase assay were used to test LUAD and LUSC.

**Results:**

LUAD and LUSC appear sharply distinct at molecular and pathological level. Let‐7a‐5p, miR‐338, miR‐375, miR‐217, miR‐627, miR‐140, miR‐147b, miR‐138‐2, miR‐584, and miR‐197 are top 10 relevant miRNAs and CLDN3, DSG3, KRT17, TMEM125, KRT5, NKX2‐1, KRT7, ABCC5, KRAS, and PLCG2 are top 10 relevant genes in NSCLC. At the same time, the miRNAs expression levels were also quite different between the two groups. Among the differential expressed miRNAs, let‐7a‐5p was significantly down‐regulated in LUAD while miR‐338 was markedly down‐regulated in LUSC. Bioinformatics analyses appeared that let‐7a‐5p directly targets high–molecular weight keratin 5 (KRT5) which were shown to be a strong risk factor for LUAD. And NK2 homeobox 1(NKX2‐1) which was associated with tumor progression in LUSC was identified as a target gene of miR‐338.

**Conclusions:**

Distinct profile of miRNAs can take a part in the development of LUAD and LUSC and thus could serve as a subtype‐specific molecular therapeutic target to protect against LUAD and LUSC.

## Graphical abstract

Non‐small cell lung cancer (NSCLC) contains 85% of lung cancer. Lung adenocarcinoma (LUAD) and lung squamous cell carcinoma (LUSC) are the largest NSCLC subgroups. The aim of the study was to investigate the underlying mechanism in developing more effective subtype‐specific molecular therapeutic procedures. Methods A total of 876 specimens were used in this study: 494 LUAD tissues (i.e., 449 LUAD tissues and 45 matched normal tissues) and 382 LUSC tissues (i.e., 337 LUSC tissues and 45 matched normal tissues). The miRNAs sequencing data were processed using R. The differential expressed miRNAs between lung cancer and normal tissues were analyzed using the limma package in R. Gene expression, western blotting, haematoxylin, eosin staining and luciferase assay were used to test LUAD and LUSC. Results LUAD and LUSC appear sharply distinct at molecular and pathological level. Let‐7a‐5p, miR‐338, miR‐375, miR‐217, miR‐627, miR‐140, miR‐147b, miR‐138‐2, miR‐584 and miR‐197 are top 10 relevant miRNAs and CLDN3, DSG3, KRT17, TMEM125, KRT5, NKX2‐1, KRT7, ABCC5, KRAS and PLCG2 are top 10 relevant genes in NSCLC. At the same time, the miRNAs expression levels were also quite different between the two groups. Among the differential expressed miRNAs, let‐7a‐5p was significantly down‐regulated in LUAD while miR‐338 was markedly downregulated in LUSC. Bioinformatics analyses appeared that let‐7a‐5p directly targets high‐molecular weight keratin 5 (KRT5) which were shown to be a strong risk factor for LUAD. And NK2 homeobox 1(NKX2‐1) which was associated with tumor progression in LUSC was identified as a target gene of miR‐338. Conclusions Distinct profile of miRNAs can take a part in the development of LUAD and LUSC, and thus could serve as a subtype‐specific molecular therapeutic target to protect against LUAD and LUSC

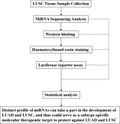

## 1 INTRODUCTION

Lung cancer is the most common cause of cancer death. It is estimated that there are 1.8 million patients worldwide, and 1.6 million cancer‐related deaths each year.^1^ Lung cancer includes small cell lung cancer (SCLC, 15%) and non‐small cell lung cancer (NSCLC, 85%), and the most common subtypes of NSCLC are lung adenocarcinoma (LUAD) and lung squamous cell carcinoma (LUSC).^2^ Although several targeted therapies have been reported to be effective, the overall survival rate of NSCLC is 20%, and the 5‐year survival rate is only 16%.^3^ Most patients have already advanced to the metastatic stage at the time of diagnosis, which is indicative of a poor prognosis. Therefore, the early detection and treatment of NSCLC are critical for understanding the development and progression of LUAD and LUSC and for identifying new disease drivers.

MicroRNAs (miRNAs) are small non‐coding RNAs of approximately 20‐22 nucleotides. They inhibit the expression of genes through post‐transcriptional mechanisms.^4^ Several studies have reported that aberrant expression of miRNAs in multiple tumor types, and miRNAs target more than 60% of all protein‐coding genes.^5^, ^6^ Mounting evidence have indicated that miRNAs play numerous regulatory roles in various cellular processes during carcinogenesis, including cell proliferation,^7^ cell migration,^8^ cell invasion,^8^ apoptosis,^9^ autophagy,^10^ and metastasis.^11^ Therefore, miRNAs hold promise as targets for individualized cancer therapy.

Although many miRNAs have been used to predict the clinical outcomes of lung carcinoma, there are many inconsistencies between studies, and the differential expression patterns of miRNAs between LUAD and LUSC tissues remain to be elucidated. The Cancer Genome Atlas Project (TCGA) is a national cancer research institute project that maintains the genetic profiles of more than 20 different types of tumors, with convenient access to raw data to all cancer researchers.^12^ The TCGA has released a considerable amount of miRNA sequencing data for patients with LUAD and LUSC. In this study, we identified differences in the expression of genes between normal lung tissues and LUAD or LUSC tissues and compared our results with miRNAs data downloaded from the TCGA. In addition, we also identified overlapping miRNAs present in both LUAD and LUSC patients and selected those with similar or different expression patterns. Using these miRNAs, we further analyzed the functions of these miRNAs. In summary, this study may provide new clues for us to understand the underlying molecular mechanisms of lung cancer and the diagnosis of LUAD and LUSC.

## 2 MATERIALS AND METHODS

### 2.1 Reagents

TaqMan miRNA probes and Power SYBR Green PCR Master Mix (cat. no. 4368577) were purchased from Applied Biosystems. Hematoxylin (cat. no. C0107), eosin (cat. no. C0109), and radioimmunoprecipitation (RIPA) buffer (cat. no. P0013B) were procured from Beyotime. For Western blotting, anti‐KRT5 (cat. no. ab52635) and anti‐NKX2‐1 (cat. no. ab76013) antibodies were purchased from Abcam. The anti‐GAPDH (cat. no sc‐25778) was purchased from Santa Cruz Biotechnology.

### 2.2 Data processing

Raw sequencing data and clinical information were downloaded from the TCGA (https://cancergenome.nih.gov/). The inclusion criteria were that each specimen had both miRNA sequencing data and clinical information. A total of 876 specimens were used in this study: 494 LUAD tissues (ie, 449 LUAD tissues and 45 matched normal tissues) and 382 LUSC tissues (ie, 337 LUSC tissues and 45 matched normal tissues). Let‐7a‐5p, miR‐338, miR‐375, miR‐217, miR‐627, miR‐140, miR‐147b, miR‐138‐2, miR‐584, and miR‐197 are top 10 relevant miRNAs and CLDN3, DSG3, KRT17, TMEM125, KRT5, NKX2‐1, KRT7, ABCC5, KRAS, and PLCG2 are top 10 relevant genes in NSCLC. The detail clinical characteristics and differential expressed miRNAs are listed in Table 1. The miRNA sequencing data were processed using R. The differential expressed miRNAs between lung cancer and normal tissues were analyzed using the limma package in R. The fold change (FC) in the expression of individual miRNAs was calculated, and differential expressed miRNAs with log2|FC|> 2.0 and those with *P* < .05 were considered to be significant.

**TABLE 1:** miRNAs differently expressed in LUAD and LUSC

Names	Number	miRNAs
LUAD_Up LUSC_Up	67	miR‐147b, miR‐9‐1, miR‐323, miR‐3653, miR‐3676, miR‐193b, miR‐188, miR‐31, miR‐33a, miR‐503, miR‐96, miR‐141, miR‐651, miR‐937, miR‐760, miR‐3648, miR‐224, miR‐3127, miR‐323b, miR‐455, miR‐590, miR‐450b, miR‐183, miR‐200b, miR‐196a‐1, miR‐576, miR‐891a, miR‐196b, miR‐301b, miR‐409, miR‐19a, miR‐130b, miR‐766, miR‐135b, miR‐708, miR‐1307, miR‐324, miR‐539, miR‐93, miR‐3200, miR‐429, miR‐424, miR‐345, miR‐200a, miR‐548v, miR‐1301, miR‐7‐1, miR‐210, miR‐21, miR‐205, miR‐450a‐1, miR‐4326, miR‐675, miR‐33b, miR‐103‐2, miR‐454, miR‐182, miR‐105‐2, miR‐940, miR‐642a, miR‐505, miR‐9‐2, miR‐1269, miR‐577, miR‐616, miR‐301a, miR‐671
LUAD_Up LUSC_Down	1	miR‐375
LUAD_Down LUSC_Down	21	miR‐138‐2, miR‐34c, miR‐143, let‐7c, miR‐133b, miR‐139, miR‐190, miR‐195, miR‐218‐2, miR‐451, miR‐133a‐1, miR‐1‐2, miR‐30a, miR‐486, miR‐1258, miR‐138‐1, miR‐1247, miR‐490, miR‐144, miR‐184, miR‐218‐1
LUAD_Up	46	miR‐217, miR‐550a‐2, miR‐450a‐2, miR‐382, miR‐425, miR‐34a, miR‐199a‐1, miR‐17, miR‐20a, miR‐331, miR‐629, miR‐660, miR‐194‐1, miR‐1287, miR‐2355, miR‐192, miR‐551a, miR‐542, miR‐19b‐1, miR‐3677, miR‐106a, miR‐148a, miR‐29b‐2, miR‐19b‐2, miR‐142, miR‐592, miR‐550a‐1, miR‐431, miR‐136, miR‐551b, miR‐199a‐2, miR‐628, miR‐29b‐1, miR‐194‐2, miR‐493, miR‐153‐2, miR‐181b‐2, miR‐154, miR‐625, miR‐653, miR‐889, miR‐487b, miR‐3607, miR‐187, miR‐199b, miR‐191
LUSC_Up	69	miR‐627, miR‐149, miR‐423, miR‐506, miR‐487a, miR‐3154, miR‐570, miR‐1293, miR‐2277, miR‐877, miR‐1291, miR‐579, miR‐129‐1, miR‐105‐1, miR‐376b, miR‐1271, miR‐3940, miR‐196a‐2, miR‐3651, miR‐1254, miR‐18a, miR‐296, miR‐1248, miR‐485, miR‐513c, miR‐203, miR‐421, miR‐412, miR‐3681, miR‐1306, miR‐1296, miR‐1224, miR‐1226, miR‐3691, miR‐615, miR‐200c, miR‐545, miR‐1245, miR‐3652, miR‐1911, miR‐2116, miR‐376a‐2, miR‐3647, miR‐494, miR‐1277, miR‐129‐2, miR‐939, miR‐329‐1, miR‐320b‐2, miR‐3682, miR‐3928, miR‐1292, miR‐3687, miR‐3610, miR‐944, miR‐1249, miR‐769, miR‐767, miR‐526b, miR‐3942, miR‐9‐3, miR‐383, miR‐3170, miR‐219‐1, miR‐483, miR‐3187, miR‐744, miR‐1270‐1, miR‐452
LUAD_Down	11	let‐7a‐3, miR‐125b‐2, let‐7a‐2, miR‐516a‐1, miR‐378, miR‐516a‐2, let‐7f‐2, miR‐378c, let‐7a‐1, miR‐584, miR‐204
LUSC_Down	25	miR‐140, miR‐497, miR‐3065, miR‐206, miR‐30b, miR‐223, miR‐552, miR‐135a‐1, miR‐145, miR‐100, miR‐338, miR‐511‐2, miR‐1976, miR‐326, miR‐511‐2, miR‐34b, miR‐3926‐1, miR‐101‐2, miR‐99a, miR‐101‐1, miR‐29c, miR‐548b, miR‐181a‐1, miR‐30d, miR‐126

### 2.3 Gene expression and Western blotting

Total RNA was isolated from tissues using TRIzol Reagent. For RT‐qPCR analysis, oligo d(T)18 primer (TaKaRa) was used for reverse transcription of mRNA. To amplify target genes, gene‐specific primer pairs (Table 2) were combined with the Power SYBR Green PCR Master Mix. The relative levels of target genes were normalized to the Gapdh level using the comparative Ct method. To amplify miRNAs, the TaqMan MicroRNA Reverse Transcription Kit and Megaplex RT Primers were used. The relative levels of miRNAs were normalized to the U6 level. For Western blotting, tissues were lysed in RIPA buffer. The proteins were resolved by sodium dodecyl‐sulfate‐polyacrylamide gel electrophoresis, transferred to a polyvinylidene fluoride membrane, and probed with the indicated antibodies.

**TABLE 2:** Gene names and sequences of primers used in this study

Gene	Forward	Reverse
ABCC5	AGTCCTGGGTATAGAAGTGTGAG	ATTCCAACGGTCGAGTTCTCC
DSG3	GCAAAAACGTGAATGGGTGAAA	TCCAGAGATTCGGTAGGTGATT
KRT17	GGTGGGTGGTGAGATCAATGT	CGCGGTTCAGTTCCTCTGTC
KRT5	CCAAGGTTGATGCACTGATGG	TGTCAGAGACATGCGTCTGC
NKX2‐1	AGCACACGACTCCGTTCTC	GCCCACTTTCTTGTAGCTTTCC
KRT7	TCCGCGAGGTCACCATTAAC	GCTCTGTCAACTCCGTCTCAT
TMEM125	CTGCTGTATCAAGTGGGTGTG	GATGTGGTCTCGTGACGCC
CLDN3	AACACCATTATCCGGGACTTCT	GCGGAGTAGACGACCTTGG
GAPDH	GGAGCGAGATCCCTCCAAAAT	GGCTGTTGTCATACTTCTCATGG

### 2.4 Hematoxylin and eosin staining

Tissues were fixed in 10% formalin, processed, embedded in paraffin, and sectioned to generate 8‐µm‐thick cross sections. Thereafter, the cross sections were stained with hematoxylin and eosin, followed by morphological observation under a light microscope.

### 2.5 Luciferase reporter assay

A 1000‐bp fragment of the KRT5 or NKX2‐1 3′‐UTR containing let‐7a‐5p or miR‐338 putative binding sites was inserted into a luciferase reporter plasmid (Genescript). A KRT5 3′‐UTR mutant was constructed by mutagenesis of the let‐7a‐5p binding site from GAGGUAG to CUCCAUC, whereas an NKX2‐1 3′‐UTR mutant was generated by mutagenesis of the miR‐338 binding site from CCAGCA to GGUCGU. The mutated 3′‐UTR fragment was inserted into an equivalent reporter plasmid (Genescript). For the luciferase reporter assays, HEK293T cells were cultured in 24‐well plates, and 0.2 μg of the firefly luciferase reporter plasmid, an equal amount of miRNA mimic or negative control RNA, and 0.2 μg of the β‐galactosidase expression plasmid were used to co‐transfect each well. The Dual‐Luciferase Reporter Assay System (Promega) was used to assay these cells at 24 hours post‐transfection.

### 2.6 Statistical analysis

All data were analyzed with GraphPad Prism 6.0 Software using two‐tailed Student's *t* tests. The expression levels of miRNAs in LUAD/LUSC specimens and matching normal tissues were analyzed using unpaired *t* tests. *P*‐values < .05 were considered statistically significant.

## 3 RESULTS

### 3.1 Transcriptomic and histopathologic differences between LUAD and LUSC tissues

**FIGURE 1:**
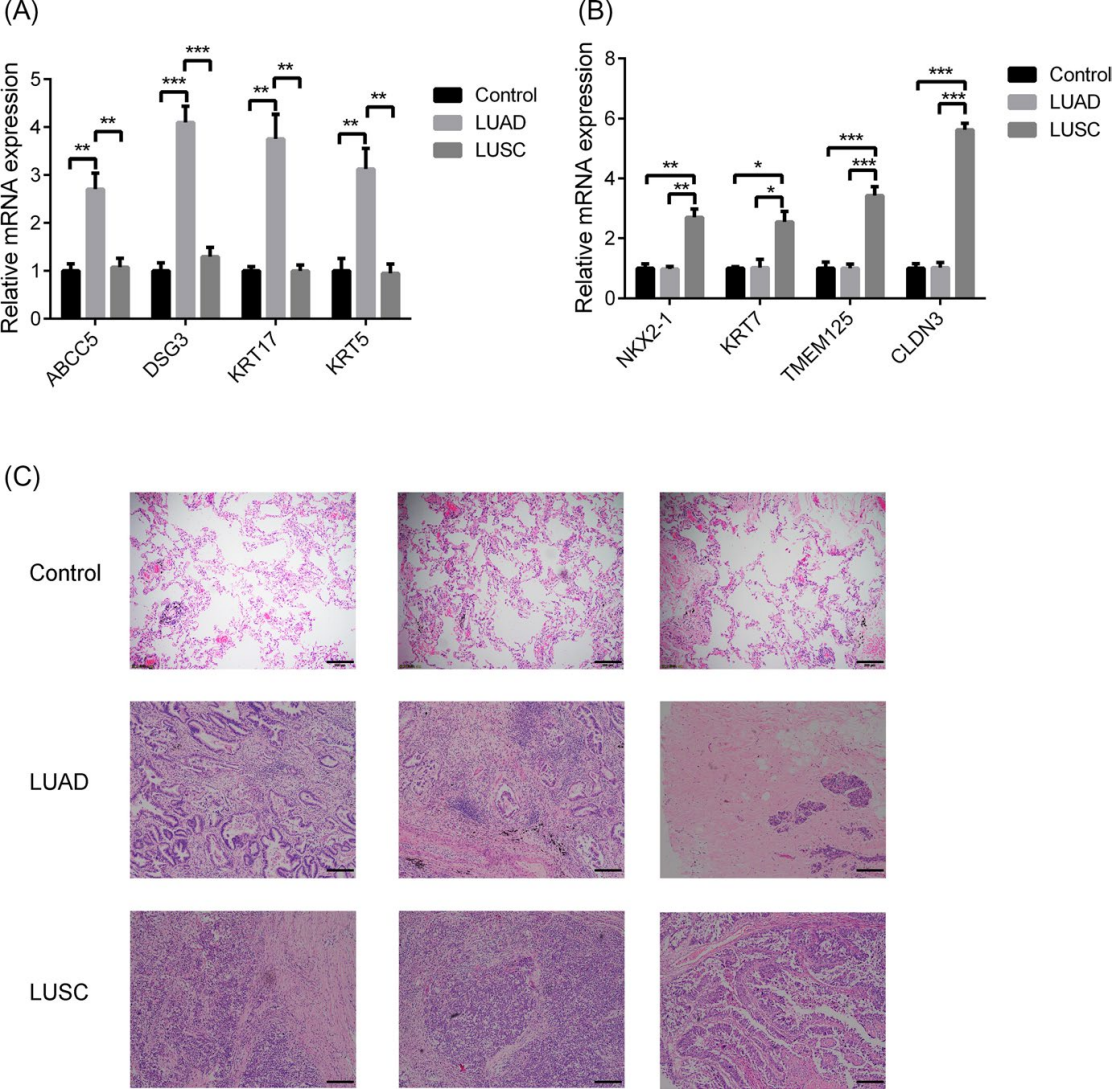
Transcriptomic and histopathologic differences between LUAD and LUSC tissues. A, Normalized expression of LUAD‐selected genes in LUAD, LUSC, and normal lung tissues. B, Normalized expression of LUSC‐selected genes in LUAD, LUSC, and normal lung tissues. C, Representative images from hematoxylin and eosin‐stained sections of LUAD, LUSC, and normal lung tissues. Scale bar: 200 μm. The data represent the mean ± SEM. *P < .05; **P < .001; ***P < .0001; (Student's *t* test)

We analyzed the transcriptome profiles and histopathology of the NSCLC subgroups. Vast differences were observed between LUAD and LUSC tissues for the two parameters. As expected, the mRNA levels of the gene sets identified as highly expressed in LUAD tissues, such as ABCC5, desmoglein 3 (DSG3), high‐molecular‐weight keratin 17 (KRT17), and keratin 5 (KRT5),^13^ were significantly increased in LUAD tissues compared with LUSC and normal lung tissues (Figure 1A). At the same time, the mRNA levels of the gene sets identified as highly expressed in LUSC tissues, such as NK2 homeobox 1 (NKX2‐1), KRT7, TMEM125, and CLDN3,^14^ were significantly increased in LUSC tissues (Figure 1B). With these consistent observations, histological analysis showed remarkable differences between LUSC and LUAD tissues (Figure 1C).

### 3.2 Identification of differentially expressed miRNAs in LUAD and LUSC tissues

**FIGURE 2:**
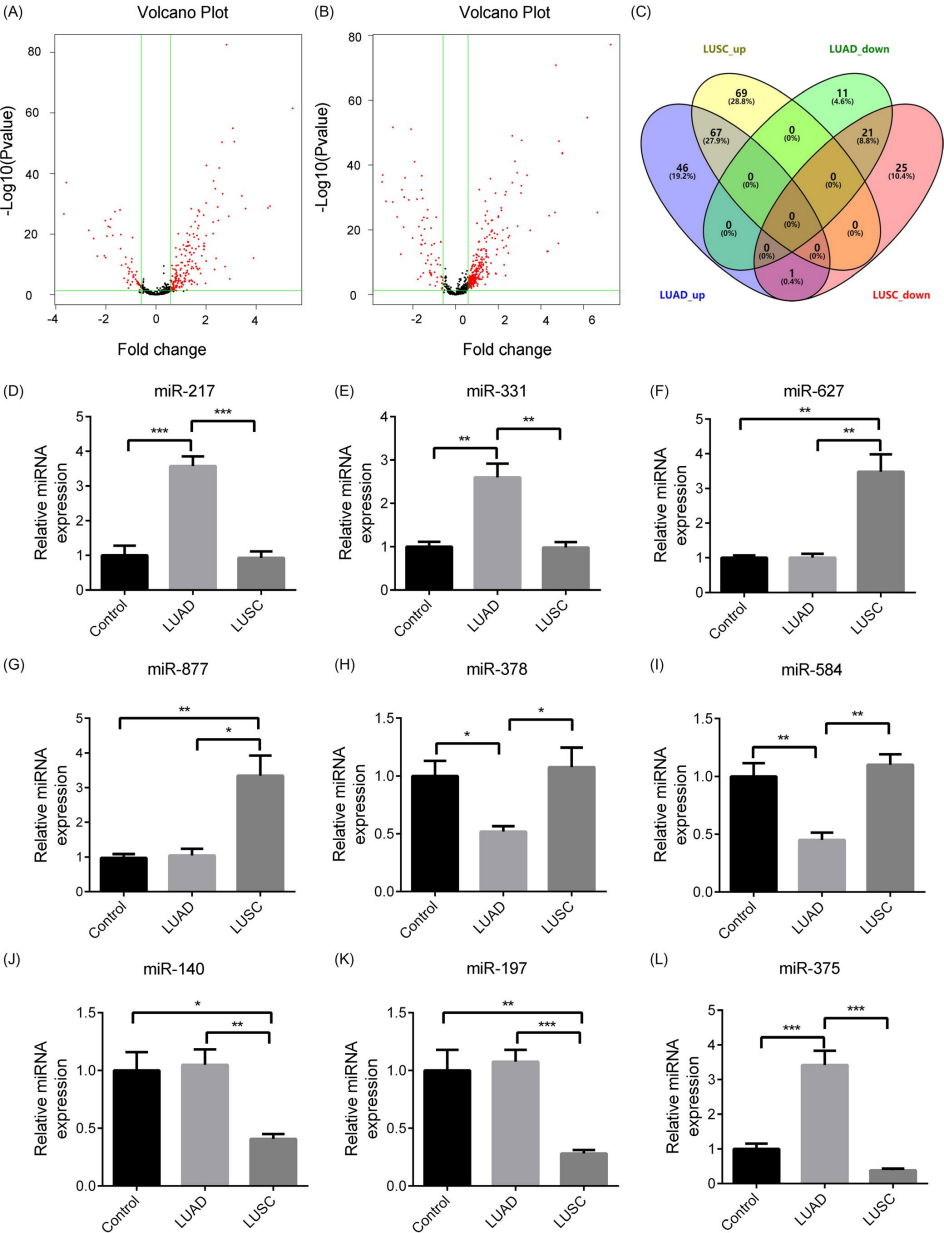
The differentially expressed miRNAs between LUAD and normal tissues. A, In the volcano plot, the red dots represent up/down‐regulated miRNAs, and black dots represent normally expressed miRNAs. B, Volcano plot graph of miRNAs in LUSC and normal lung tissues. C, Venn diagram represents miRNAs whose expression was different among LUAD, LUSC, and normal lung tissues. A‐I, Relative levels of miR‐217 (A), miR‐331 (B), miR‐627 (C), miR‐877 (D), miR‐378 (E), miR‐584 (F), miR‐140 (G), miR‐197 (H), and miR‐375 (I). The data represent the mean ± SEM. *P < .05; **P < .001; ***P < .0001; (Student's *t* test)

A total of 494 LUAD tissues were collected for analysis, including 449 lung adenocarcinoma tissues and 45 matched normal tissues. Detailed clinical features were recorded, including the age at diagnosis, metastasis, lymph node status, stage, T stage, and smoking history (Table 3). The cutoff criteria (ie, *P* < .05 and |log2FC|> 2.0) identified 146 differentially expressed miRNAs, including 114 up‐regulated miRNAs and 32 down‐regulated miRNAs. To validate that the *P*‐value and |log2FC| cutoff criteria were valid, we presented these results as a volcano plot **(**Figure 2A).

**TABLE 3:** Clinical characteristics of lung adenocarcinoma patients

Variables	Case, n (%)
Age at diagnosis	
<60	119 (26.5)
≥60	302 (67.3)
NA	28 (6.2)
Metastasis	
M0	287 (63.9)
M1	19 (4.2)
MX	139 (31.0)
NA	4 (0.9)
Lymph node status	
N0	292 (65.0)
N1‐3	147 (32.7)
NX	9 (2.0)
NA	1 (0.2)
Stage	
I + II	352 (78.4)
III + IV	93 (20.7)
NA	4 (0.9)
T stage	
T1 + T2	391 (87.1)
T3 + T4	55 (12.2)
TX	3 (0.7)
Smoking history category	
<3	170 (37.9)
≥3	265 (59.0)
NA	14 (3.1)

Abbreviation: NA, not available.

To assess the differential expressed miRNAs in LUSC tissues, a total of 382 LUSC specimens were included in this study, in which 337 were LUSC tissues and 45 were normal lung tissues. Detailed clinical features were recorded, including the age at diagnosis, metastasis, lymph node status, stage, T stage, and smoking history (Table 4). The cutoff criteria (ie, *P* < .05 and |log2FC|> 2.0) identified 183 differential expressed miRNAs. Among these, 136 genes were up‐regulated and 47 genes were down‐regulated. To validate that the *P*‐value and |log2FC| cutoff criteria were valid, we presented these results as a volcano plot (Figure 2B). Venn diagrams were prepared to compare the differential expressed miRNAs identified in the LUAD and LUSC tissues (Figure 2C). A total of 46 miRNAs were found to be up‐regulated in LUAD tissues, whereas 69 miRNAs were found to be up‐regulated in LUSC tissues (Table 1). As shown in Table 1, 11 miRNAs were down‐regulated in LUAD tissues, and 25 miRNAs were down‐regulated in LUSC tissues. Further studies are needed as the differential expressed miRNAs in LUAD and LUSC tissues may play important roles in the development of different types of lung cancer.

**TABLE 4:** Clinical characteristics of lung squamous cell carcinoma patients. NA, not available

Variables	Case, n (%)
Age at diagnosis	
<60	60 (17.8)
≥60	270 (80.1)
NA	7 (2.1)
Metastasis	
M0	257 (76.3)
M1	3 (0.9)
MX	77 (22.8)
Lymph node status	
N0	216 (64.1)
N1‐3	116 (34.4)
NX	5 (1.5)
Stage	
I + II	280 (83.1)
III + IV	54 (16.0)
NA	3 (0.9)
T stage	
T1 + T2	268 (79.5)
T3 + T4	69 (20.5)
Smoking history category	
<3	116 (34.4)
≥3	210 (62.3)
NA	11 (3.3)

### 3.3 Validation of candidate miRNAs by RT‐qPCR

To validate our results, we selected nine miRNAs and performed RT‐qPCR using 20 LUAD tissues, 23 LUSC tissues, and 15 normal lung tissues. The nine miRNAs were selected from five groups, namely the LUAD up‐regulation group, LUAD down‐regulation group, LUSC up‐regulation group, LUSC down‐regulation group, and LUAD up‐regulation/LUSC down‐regulation group. The results showed that the different expression levels of these nine miRNAs were in agreement with our results, indicating that our analysis was reliable (Figure 2D‐L).

### 3.4 Let‐7a‐5p may prevent LUAD by inhibiting KRT5

**FIGURE 3:**
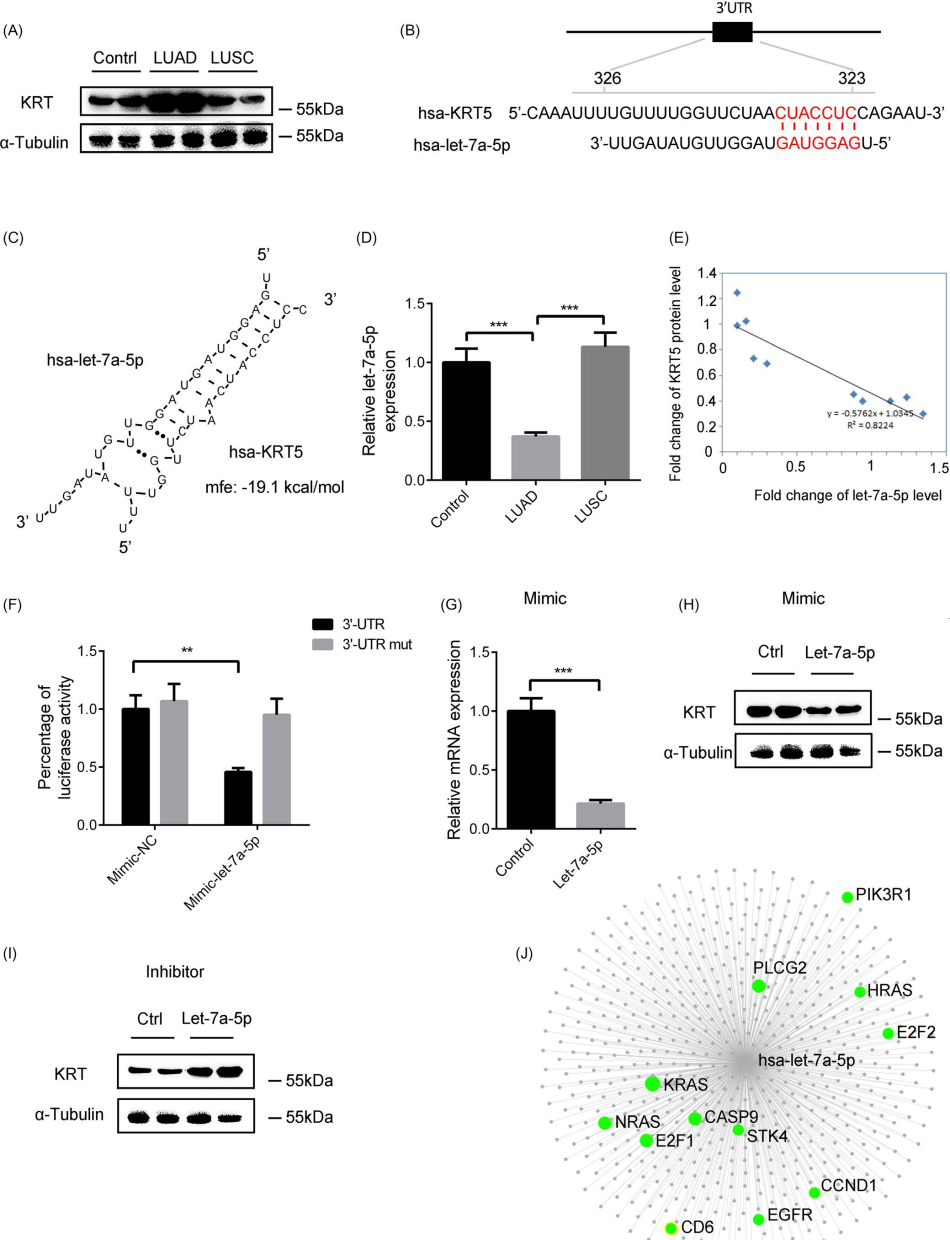
Let‐7a‐5p may prevent LUAD development by inhibiting KRT5. A, KRT5 protein levels in LUAD, LUSC, and normal lung tissues. B, Putative miRNA target sites of let‐7a‐5p within the 3′‐UTR of KRT5. C, Bioinformatic prediction of let‐7a‐5p target sites and free energy values within the 3′‐UTR of the human KRT5 gene. D, Relative expression level of let‐7a‐5p in LUAD, LUSC, and normal lung tissues. E, Pearson's correlation scatter plot of the fold changes of let‐7a‐5p and KRT5 protein in human LUAD tissue pairs. The data represent the mean ± SEM. *P < .05; **P < .001; ***P < .0001; (Student's *t* test). F, Relative luciferase activity in HEK293T cells transfected with plasmid reporter constructs containing the 3′‐UTR or mutated 3′‐UTR of KRT5, co‐transfected with mimic‐let‐7a‐5p. G, H, KRT5 mRNA (G) and protein (H) levels in HEK293T cells transfected with let‐7a‐5p mimic. I, KRT5 protein levels in HEK293T cells transfected with let‐7a‐5p antisense oligonucleotide. J, miRNA‐target interactions and functional associations using network‐based visual analysis

Previously, results have showed that KRT5 may play an important role in the development of LUAD; we found that the KRT5 mRNA level was increased in LUAD tissues compared with LUSC and normal lung tissues (Figure 1A). Similarly, the KRT5 protein level was significantly up‐regulated in LUAD tissues (Figure 3A). This prompted us to hypothesize that one of the regulatory factors may be a miRNA. We searched the TargetScan database to identify miRNAs that could target the 3′‐ untranslated region (3′‐UTR) of the human KRT5 gene. We found that let‐7a‐5p, which has a conserved target site in the 3′‐UTR of KRT5, was down‐regulated in LUAD (Figure 3B,C). RT‐qPCR confirmed that the expression of let‐7a‐5p was markedly decreased in LUAD tissues (Figure 3D). As shown in Figure 3E, let‐7a‐5p expression was highly inversely correlated with KRT5 expression. Thereafter, the reporter plasmid containing the KRT5 3′‐UTR were used to transfect human embryonic kidney 293 T (HEK293T) cells, which showed significantly decreased luciferase activity in the presence of ectopic let‐7a‐5p. When the conserved seed sequence was mutated, the miRNA‐induced repression of the KRT5 3′‐UTR was abrogated (Figure 3F). RT‐qPCR and Western blotting analysis showed that let‐7a‐5p mimic reduced both KRT5 mRNA and protein levels (Figure 3G,H). Conversely, the inhibition of let‐7a‐5p using an antisense oligonucleotide markedly increased the KRT5 protein level (Figure 3I). As miRNAs can post‐transcriptional regulate the expression of target genes, miRNA‐target interactions and functional associations were analyzed through network‐based visual analysis (miRNet). The highlighted genes were found to associate with NSCLC (Figure 3J). Taken together, these results showed that a decreased level of let‐7a‐5p may be involved in the development of LUAD by targeting KRT5.

### 3.5 MiR‐338 may prevent LUSC by inhibiting NKX2‐1

**FIGURE 4:**
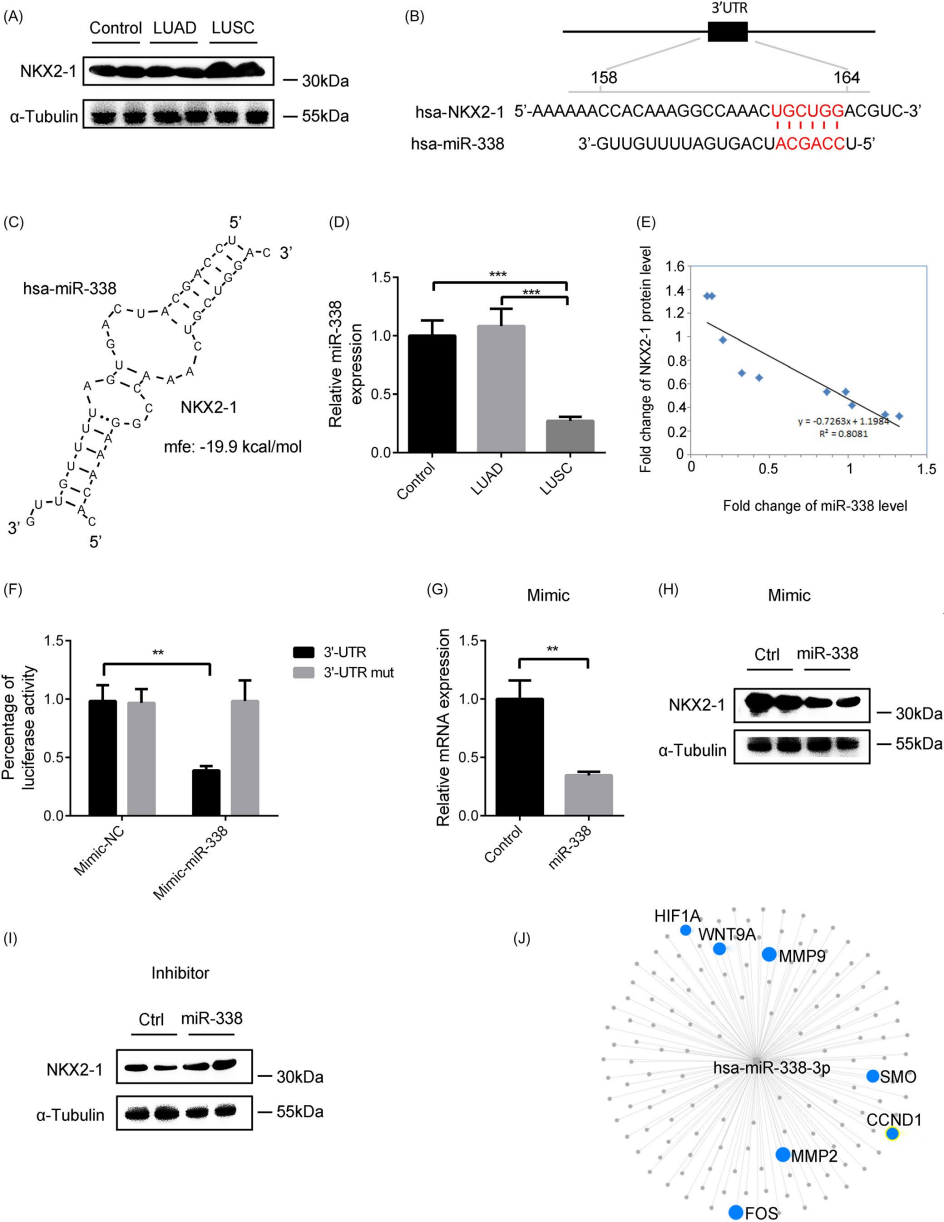
MiR‐338 may prevent LUSC development by inhibiting NKX2‐1. A, NKX2‐1 protein levels in LUSC, LUSC, and normal lung tissues. B, Putative miRNA target sites of miR‐338 within the 3′‐UTR of NKX2‐1. C, Bioinformatic prediction of miR‐338 target sites and free energy values within the 3′‐UTR of the human NKX2‐1 gene. D, Relative expression level of miR‐338 in LUSC, LUSC, and normal lung tissues. E, Pearson's correlation scatter plot of the fold changes of miR‐338 and NKX2‐1 protein in human LUSC tissue pairs. The data represent the mean ± SEM. *P < .05; **P < .001; ***P < .0001; (Student's *t* test). F, Relative luciferase activity in HEK293T cells transfected with plasmid reporter constructs containing the 3′‐UTR or mutated 3′‐UTR of NKX2‐1, co‐transfected with mimic‐miR‐338. G, H, NKX2‐1 mRNA (G) and protein (H) levels in HEK293T cells transfected with miR‐338 mimic. I, NKX2‐1 protein levels in HEK293T cells transfected with miR‐338 antisense oligonucleotide. J, miRNA‐target interactions and functional associations using network‐based visual analysis

Also, previously results showed that NKX2‐1 may play an important role in the development of LUSC, we found that the NKX2‐1 mRNA level was markedly increased in LUAD (Figure 1B). At the same time, the NKX2‐1 protein level was significantly up‐regulated (Figure 4A). We also searched the TargetScan database to identify miRNAs that could target the 3′‐UTR of the human NKX2‐1 gene. We found that miR‐338, which has a conserved target site in the 3′‐UTR of NKX2‐1, was down‐regulated in LUAD (Figure 4B,C). RT‐qPCR verified that the miR‐338 level was significantly decreased in LUAD (Figure 4D). As shown in Figure 4E, miR‐338 expression was highly inversely correlated with NKX2‐1 expression. Thereafter, the reporter plasmid containing the NKX2‐1 3′‐UTR were used to transfect HEK293T cells, and significantly decreased luciferase activity in the presence of ectopic miR‐338 was observed. When the conserved seed sequence was mutated, the miRNA‐induced repression of the NKX2‐1 3′‐UTR was abrogated (Figure 4F). RT‐qPCR and Western blotting analysis showed that miR‐338 mimic reduced both NKX2‐1 mRNA and protein levels (Figure 4G,H). Conversely, inhibition of miR‐338 using an antisense oligonucleotide increased the NKX2‐1 protein level (Figure 4I). miRNA‐target interactions and functional associations were also analyzed through miRNet. The highlighted genes were found to associate with cancer (Figure 4J). In summary, these results show that a decreased level of miR‐338 may be involved in the development of LUAD by targeting NKX2‐1.

## 4 DISCUSSION

With the introduction of molecular‐targeted therapies, such as gefitinib and erlotinib,^15^ the NSCLC mortality rate has dramatically declined in recent decades. However, unlike SCLC, the proliferation of NSCLC is slower, as is its metastasis, so most NSCLC patients are diagnosed at middle/late stages of the disease.^16^ If tumor behavior could be reliably predicted at an early stage, the prognosis of lung cancer patients would be greatly improved. Therefore, it is critical to understand the molecular mechanisms of lung cancer development and to identify new biomarkers for early diagnosis. In this study, 329 differential expressed miRNAs were identified in LUAD and LUSC tissues, and we compared the differential expressed overlapping miRNAs between cancers. According to the Venn diagram, miR‐210 and miR‐9‐1 were up‐regulated in LUAD and LUSC tissues, whereas miR‐144 and miR‐486 were down‐regulated in both types of cancer. miR‐375 was up‐regulated in LUAD specimens but down‐regulated in LUSC specimens.

Previous studies have reported that many miRNAs can function as onco‐promotive or onco‐suppressive miRNAs by inhibiting oncogenes or tumor suppressor genes, respectively. This phenomenon is characteristic of nearly all types of cancer.^17^, ^18^. These miRNAs regulate gene expression by hybridizing to complementary sequences in the 3′‐UTR or other regions of the target mRNA. Subsequently, this gene is translated by mRNA and functions by destabilizing or degrading mRNA.^19^ These RNAs play essential roles in NSCLC development by regulating various processes, including transcription, gene expression, proliferation, and apoptosis.^20^, ^21^, ^22^, ^23^

A considerable number of miRNAs have been reported to associate with NSCLC; these include miR‐206,^24^ miR‐335‐5p,^25^ miR‐30a‐5p,^26^ miR‐205,^27^ miR‐29b,^28^ miR‐847,^29^ miR‐449c,^30^ miR‐138,^31^ miR‐100,^32^ and miR‐155.^33^ However, many previous studies have employed few specimens, different specimen types, different detection platforms, and different detection methods; many have also evaluated a relatively limited number of miRNAs. In the present study, we analyzed high‐throughput data and identified several miRNAs that may play important roles in regulating the development of LUAD or LUSC. Some of the miRNAs have already been shown to strongly correlate with lung cancer. For example, Puisségur et al (2011) showed that miR‐210, which is overexpressed during the late stages of lung cancer, can facilitate mitochondrial alterations that associate with the modulation of HIF‐1 activity.^34^ In another study, Grosso et al^35^ reported that miR‐210 can promote hypoxia and increase the radiation resistance of human lung cancer cell lines. Furthermore, Chen et al^36^ found that miR‐9‐1 decreased FoxO1 expression by directly curbing its mRNA translation. They also showed that miR‐9‐1 has a substantial impact on tumor growth.

The two miRNAs that were found to be down‐regulated in both LUAD and LUSC tissues (ie, miR‐144 and miR‐486) have been identified as tumor suppressors in different studies. Chen et al^37^ suggested that miR‐144 inhibits lung cancer cell proliferation and induces cell apoptosis and autophagy by targeting TIGAR. In addition, data from Song et al^38^ showed that enhanced miR‐144 expression can improve the radiosensitivity of NSCLC by targeting ATF2, thus representing a potentially new therapeutic target for NSCLC. Peng et al^39^ demonstrated that miR‐486 is capable of regulating insulin growth factors, reducing cancer cell growth and migration, and inducing apoptosis in NSCLC.

The miRNAs differential expressed in LUAD and LUSC tissues may play important roles in regulating the development of LUAD and LUSC. In this study, we found that let‐7a‐5p may prevent LUAD by inhibiting KRT5 expression, which was previously found to be up‐regulated in LUAD. At the same time, miR‐338 may prevent LUSC by inhibiting NKX2‐1 expression. These findings were interesting as both let‐7a‐5p and miR‐338 were down‐regulated in LUAD and LUSC tissues in this study. Therefore, it is important to determine whether the differential expressed miRNAs in LUAD and LUSC tissues identified in this study create a network that can induce the development of a different type of NSCLC.

Taken collectively, we identified several miRNAs as potential biomarkers and targets for NSCLC. Further studies are needed to validate our findings using a larger sample size. Additional functional studies are also required to explore the molecular mechanisms of these miRNAs in the development and progression of lung cancer.
